# A role for endoplasmic reticulum exit sites in foot-and-mouth disease virus infection

**DOI:** 10.1099/vir.0.055442-0

**Published:** 2013-12

**Authors:** Rebecca Midgley, Katy Moffat, Stephen Berryman, Philippa Hawes, Jennifer Simpson, Daniel Fullen, David. J. Stephens, Alison Burman, Terry Jackson

**Affiliations:** 1 The Pirbright Institute, Pirbright, Surrey GU24 0NF, UK; 2 Cell Biology Laboratories, School of Biochemistry, Medical Sciences Building, University of Bristol, University Walk, Bristol BS8 1TD, UK

## Abstract

Picornaviruses replicate their genomes in association with cellular membranes. While enteroviruses are believed to utilize membranes of the early secretory pathway, the origin of the membranes used by foot-and-mouth disease virus (FMDV) for replication are unknown. Secretory-vesicle traffic through the early secretory pathway is mediated by the sequential acquisition of two distinct membrane coat complexes, COPII and COPI, and requires the coordinated actions of Sar1, Arf1 and Rab proteins. Sar1 is essential for generating COPII vesicles at endoplasmic reticulum (ER) exit sites (ERESs), while Arf1 and Rab1 are required for subsequent vesicle transport by COPI vesicles. In the present study, we have provided evidence that FMDV requires pre-Golgi membranes of the early secretory pathway for infection. Small interfering RNA depletion of Sar1 or expression of a dominant-negative (DN) mutant of Sar1a inhibited FMDV infection. In contrast, a dominant-active mutant of Sar1a, which allowed COPII vesicle formation but inhibited the secretory pathway by stabilizing COPII coats, caused major disruption to the ER–Golgi intermediate compartment (ERGIC) but did not inhibit infection. Treatment of cells with brefeldin A, or expression of DN mutants of Arf1 and Rab1a, disrupted the Golgi and enhanced FMDV infection. These results show that reagents that block the early secretory pathway at ERESs have an inhibitory effect on FMDV infection, while reagents that block the early secretory pathway immediately after ER exit but before the ERGIC and Golgi make infection more favourable. Together, these observations argue for a role for Sar1 in FMDV infection and that initial virus replication takes place on membranes that are formed at ERESs.

## Introduction

Foot-and-mouth disease (FMD) is one of the most economically important viral diseases of domestic livestock affecting cattle, sheep, goats and pigs (Scudamore & Harris, 2002). The aetiological agent, FMD virus (FMDV) is the type species of the genus *Aphthovirus* within the family *Picornaviridae*, a family of non-enveloped, single-stranded, positive-sense RNA viruses, which includes other important viruses of man and animals such as poliovirus (PV) coxsackieviruses (CV) and swine vesicular disease virus. Genome replication of positive-sense RNA viruses takes place on cellular membranes (Gazina *et al.*, 2002; Hsu *et al.*, 2010). For most picornaviruses, the precise origin of these membranes is unclear, but viruses of the genus *Enterovirus* of the family *Picornaviridae* (e.g. PV and CVB3) are believed to utilize membranes from the early secretory pathway for replication (Hsu *et al.*, 2010; Rust *et al.*, 2001).

The early secretory pathway consists of the endoplasmic reticulum (ER), the ER–Golgi intermediate compartment (ERGIC) and the Golgi, and the transport vesicles that shuttle between them. The ERGIC is the first compartment after the ER and serves as the initial site of protein sorting (Appenzeller-Herzog & Hauri, 2006; Hauri *et al.*, 2000). The Golgi is organized as a series of cisternae including the *cis*-, medial- and *trans*-Golgi networks and is central for sorting and packaging of macromolecules for delivery to endosomes, the plasma membrane or the cell exterior (Altan-Bonnet *et al.*, 2004). Vesicle trafficking through the early secretory pathway is mediated by the sequential acquisition of two distinct membrane coat complexes, COPII (coat protein II) and COPI and requires the coordinated actions of Sar1, Arf1 and Rab proteins (Duden, 2003; Stephens *et al.*, 2000). Traffic between the ER and ERGIC is mediated by COPII-coated vesicles, which form at discrete sites on the ER called ER exit sites (ERESs). The small GTPase, Sar1, is essential for COPII formation (Hughes & Stephens, 2008) and is recruited and activated at ERES by Sec12, a Sar1-specific guanine-nucleotide exchange factor (GEF). Activated Sar1 initiates vesicle formation by recruiting the inner COPII coat components Sec23 and Sec24. Recruitment of the outer coat components (Sec13/Sec31) follows and the mature coated vesicles bud from the ER. Sec23 is the GTPase activating protein (GAP) for Sar1. Consequently, Sar1 is converted to its inactive GDP-bound form and COPII coats rapidly dissociate from the vesicles (Stephens *et al.*, 2000), which then acquire COPI in a process known as COPII/COPI exchange before fusion with the ERGIC. COPI-coated vesicles also mediate secretory-vesicle traffic from the ERGIC to the Golgi, and retrograde transport from the ERGIC and Golgi to the ER (Beck *et al.*, 2009). COPI coat formation requires the GTPase ADP-ribosylation factor 1 (Arf1). Arf1 is activated on the Golgi by two related GEFs called GBF1 and BIGs. GBF1 is the only known Arf1–GEF localized to the *cis*-Golgi and is required for transport-vesicle trafficking between the ER and Golgi (Alvarez *et al.*, 2003; Claude *et al.*, 1999; Kawamoto *et al.*, 2002), whereas BIGs are responsible for Arf1 recruitment on the *trans*-Golgi (Manolea *et al.*, 2008). Rab proteins also regulate membrane trafficking through the secretory pathway (Schwartz *et al.*, 2007; Stenmark, 2009) and function in vesicle formation, transport, tethering, docking and membrane fusion, and maintenance of secretory organelle structure (Pfeffer, 2001; Zerial & McBride, 2001). For example, Rab1 isoforms are localized to Golgi membranes and required for ER to Golgi transport (Dumaresq-Doiron *et al.*, 2010; Monetta *et al.*, 2007; Plutner *et al.*, 1991).

Recent studies have provided evidence that PV and CVs generate membranes for replication by subversion of Arf1-dependent COPI vesicle formation (Belov *et al.*, 2007, 2010; Hsu *et al.*, 2010; Lanke *et al.*, 2009; Teterina *et al.*, 2011; Wessels *et al.*, 2006a, b). PV 3A protein binds GBF1 and modulates recruitment of Arf1 effectors to favour phosphatidylinositol 4-kinase (PI4K) over COPI components. This creates membranes that are devoid of COPI and enriched for phosphatidylinositol 4-phosphate, which promote membrane binding of the viral RNA-dependent RNA polymerases and formation of the viral replication complex. Hence, brefeldin A (BFA), which inhibits GBF1, or small interfering RNA (siRNA) depletion of Arf1 or GBF1 inhibits enterovirus replication (Hsu *et al.*, 2010; Mossessova *et al.*, 2003; Peyroche *et al.*, 1999; Renault *et al.*, 2003). PV has also been implicated in subverting COPII vesicles to provide replication membranes (Rust *et al.*, 2001) and more recently, PV infection has been reported to transiently stimulate COPII vesicle production, but it is not established if this is beneficial for the host cell or virus (Trahey *et al.*, 2012). For enterovirus 71 (a BFA-sensitive picornavirus), COPI but not COPII has been shown to be required for infection (Wang *et al.*, 2012).

Two observations point to significant differences between FMDV and enteroviruses in their interactions with the early secretory pathway. First, FMDV and PV inhibit protein secretion by different mechanisms (Choe *et al.*, 2005; Moffat *et al.*, 2005, 2007). The PV 3A protein inhibits protein secretion, whereas for FMDV secretion is not blocked by 3A but instead by 2B and 2C. Secondly, BFA inhibits PV but not FMDV replication (Gazina *et al.*, 2002; Martin-Acebes *et al.*, 2008; Monaghan *et al.*, 2004; O’Donnell *et al.*, 2001), suggesting that FMDV modifies cellular membranes for replication in a GBF1- and Arf1-independent process. In this report, we investigated the role of Sar1, Arf1 and Rab proteins in FMDV infection using BFA-sensitive bovine enterovirus (BEV) as a comparison and have provided evidence that the membranes used for FMDV infection most likely derive from ERESs.

## Results and Discussion

### FMDV disrupts membranes of the early secretory pathway

**Fig. 1.**
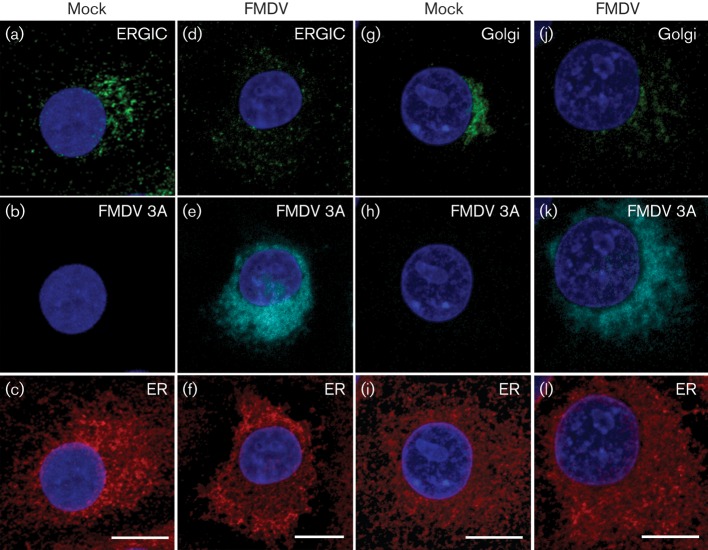
FMDV disrupts membranes of the ERGIC and Golgi. IBRS2 cells were mock-infected (mock) or infected (m.o.i. 0.5) with FMDV for 3 h and processed for confocal microscopy. (a–c) A typical mock-infected cell co-labelled for the ERGIC (ERGIC-53: green), FMDV 3A (using 2C2: cyan) and ER (ERp57: red). (d–f) An FMDV-infected cell labelled as in (a–c). (g–i) A typical mock-infected cell co-labelled for the Golgi (Giantin: green), FMDV 3A (2C2: cyan) and ER (ERp57: red). (j–l) An FMDV-infected cell labelled as in (g–i). Cell nuclei are shown in blue. Each image shows a projection of 14 sections; spacing 0.5 μm. Bars, 10 μm.

Fig. 1 shows labelling for the ER, ERGIC and Golgi in mock- and FMDV-infected IBRS2 cells. In infected cells the ER remained largely intact compared with the mock-infected cells (Fig. 1c, f, i and l) while, consistent with previous reports (Martin-Acebes *et al.*, 2008; Monaghan *et al.*, 2004), the Golgi was disrupted (Fig. 1g, j). The ERGIC consists of tubulovesicular clusters that occupy a characteristic perinuclear location close to the Golgi and additional punctae distributed throughout the cell periphery (Bannykh *et al.*, 1996; Klumperman *et al.*, 1998; Schweizer *et al.*, 1988). In infected cells, the characteristic perinuclear ERGIC clustering was lost whereas labelling at the cell periphery appeared unchanged (Fig. 1a, d). Virtually all of the infected cells showed a similar labelling pattern including those with an apparent low level of FMDV infection (i.e. low labelling for viral proteins), which suggests that the ERGIC and Golgi are disrupted early after infection and before major changes are detected in the ER.

### BFA disrupts the ERGIC and Golgi and enhances FMDV infection

**Fig. 2.**
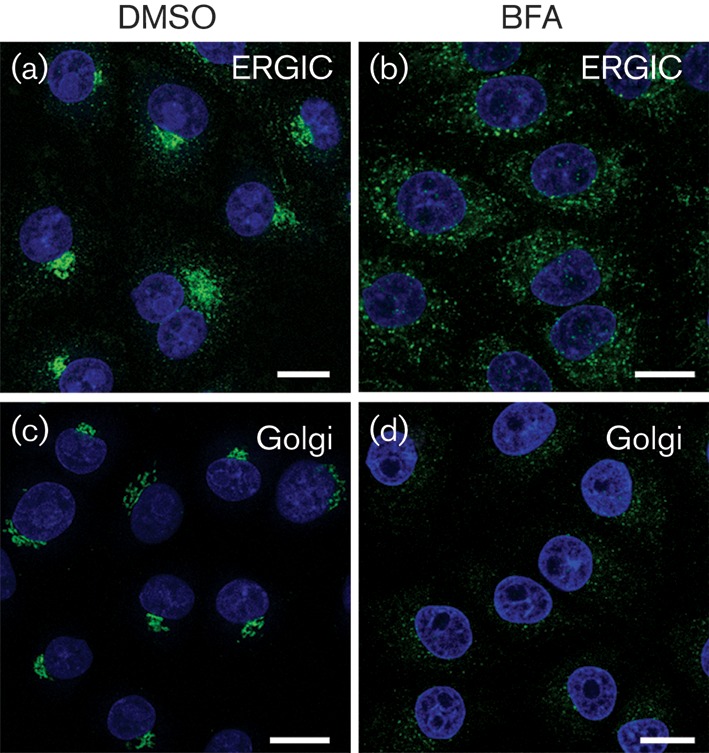
BFA disrupts the ERGIC and Golgi in IBRS2 cells. IBRS2 cells were treated with DMSO (mock) or BFA (5 μg ml^−1^) for 0.5 h. (a, b) Mock-treated (a) and BFA-treated (b) cells labelled for ERGIC (ERGIC-53; green). (c, d) Mock-treated (c) and BFA-treated (d) cells labelled for Golgi (GM130; green). Nuclei are shown in blue. Bars, 10 μm.

BFA arrests membrane flow through the secretory pathway resulting in disruption of the ERGIC and Golgi (Claude *et al.*, 1999; Dascher & Balch, 1994; Fujiwara *et al.*, 1988; Lippincott-Schwartz *et al.*, 1989; Togawa *et al.*, 1999). Treatment of IBRS2 cells with BFA did not appear to perturb the ER (data not shown). In contrast, BFA disrupted the ERGIC (Fig. 2a, b) and Golgi (Fig. 2c, d). Punctate labelling for the ERGIC was present throughout the cytosol, but the characteristic perinuclear clusters were lost (Fig. 2b) and the Golgi showed extensive fragmentation (Fig. 2d).

**Fig. 3.**
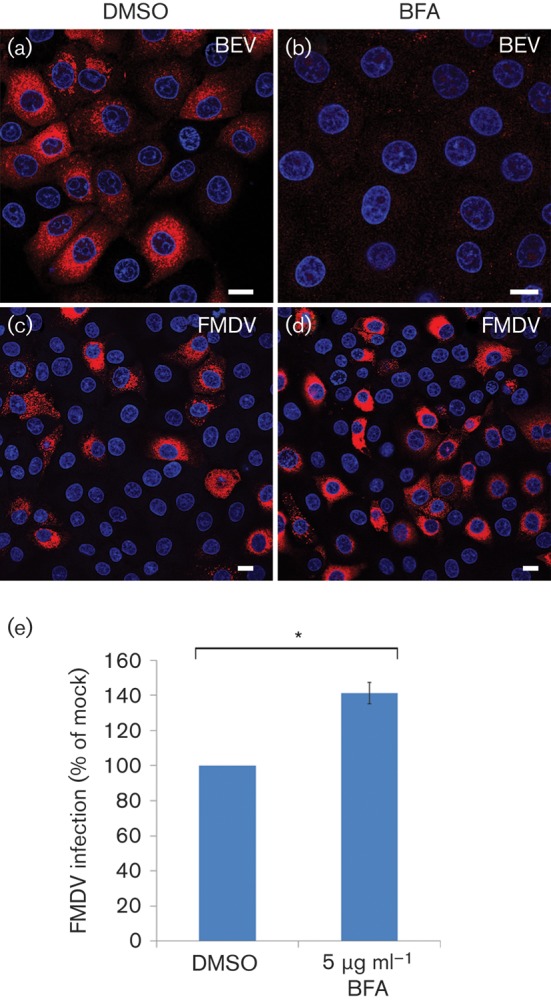
BFA enhances FMDV infection. (a–d) IBRS2 cells were mock-treated with DMSO (a, c) or BFA (5 μg ml^−1^; b, d) for 0.5 h and infected with BEV (m.o.i 1.0) or FMDV (m.o.i. 0.3) for 3.5 h and processed for confocal microscopy using virus-specific antisera. Infected cells are labelled red. Nuclei are shown in blue. Bars, 10 μm. (e) Percentage of BFA-treated cells infected by FMDV normalized to cells treated with DMSO. The mean±sem is shown for triplicate experiments counting ≥750 cells per coverslip. Student’s *t*-test was used to determine statistical significance (*P<0.01).

Most picornaviruses, including enteroviruses, are sensitive to BFA, whereas FMDV is unusual among the picornaviruses in being resistant to this reagent (Martin-Acebes *et al.*, 2008; Maynell *et al.*, 1992; Monaghan *et al.*, 2004; O’Donnell *et al.*, 2001). Fig. 3 shows the effects of BFA on infection of IBRS-2 cells by FMDV and BEV. In this experiment, cells were mock treated with DMSO or treated with BFA to induce disruption of the ERGIC and Golgi before infection. At the end of the experiment, the cells were labelled for FMDV or BEV using virus-specific antisera. Fig. 3(a, b) confirms that BFA inhibited BEV infection. BFA is reported to have little or no effect on FMDV yields (Martin-Acebes *et al.*, 2008; Monaghan *et al.*, 2004; O’Donnell *et al.*, 2001). However, Martin-Acebes *et al.* (2008) reported an ~25 % increase in the number of infected cells following BFA treatment. Therefore, we investigated the effects of BFA on FMDV using a low m.o.i. Fig. 3(c–e) shows that BFA treatment resulted in an ~40 % increase in the proportion of cells infected compared with mock-treated cells. Together, the above results confirmed that BFA disrupts the ERGIC and Golgi and showed that FMDV infection does not require these organelles to be intact. Furthermore, BFA resulted in an apparent increase in infection by FMDV.

### FMDV infection is enhanced by dominant-negative (DN) Arf1

**Fig. 4.**
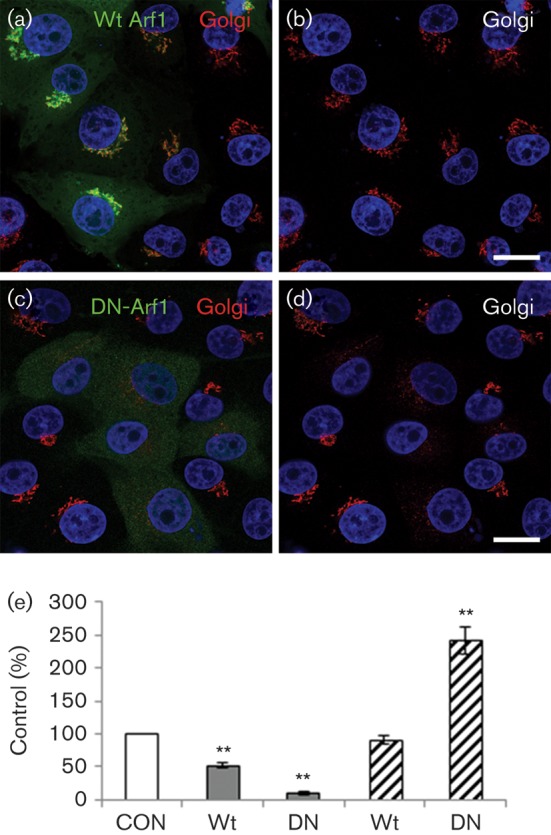
DN-Arf1 enhances infection by FMDV but not BEV. IBRS2 cells (on coverslips) were transfected with either wt Arf1 (GFP-wt-Arf1) or DN-Arf1 (HA-DN-Arf1^T31N^). (a) Cells transfected with GFP-wt-Arf1 (green) labelled for the Golgi (GM130, red). (b) The same cells as in (a) showing Golgi labelling only. (c) Cells transfected with HA-DN-Arf1^T31N^ labelled for the HA tag (green) and Golgi (GM130, red). (d) The same cells as in (c) showing Golgi labelling only. The cell nuclei are shown in blue. Bars, 10 μm. (e) Cells were transfected with GFP-wt-Arf1 (wt) or HA-DN-Arf1^T31N^ (DN) and infected with BEV (grey bars) or FMDV (hatched bars) (m.o.i. 0.3) for 3.5 h. The level of infection of cells positive for an Arf1 transgene was normalized to the non-expressing cells (control, CON) of the same coverslip. The data show the mean±sem for three independent experiments, each carried out with triplicate samples (*n*≥500 cells per coverslip). Student’s *t*-test was used to determine statistical significance (**P<0.01).

BFA causes Golgi disruption and inhibits enterovirus replication by stabilizing the complex between GDP–Arf1 and GBF1 (Dascher & Balch, 1994; Mossessova *et al.*, 2003; Peyroche *et al.*, 1999; Renault *et al.*, 2003). DN mutants of Arf1 have similar effects to BFA and cause the Golgi to collapse (Dascher & Balch, 1994), and therefore would be expected to inhibit enterovirus infections. To investigate a role for Arf1 we used transient transfection of GFP-tagged wt Arf1 (GFP-wt-Arf1) and haemagglutinin (HA)-tagged DN-Arf1 (HA-DN-Arf1^T31N^). Transfected cells were processed for confocal microscopy and the cells expressing a transgene were identified by either the GFP or the HA tag. Fig. 4 shows that, in agreement with previous observations, wt Arf1 co-localized with the Golgi (Fig. 4a, b), while DN-Arf1 caused extensive Golgi disruption (Fig. 4c, d) (Dascher & Balch, 1994; Honda *et al.*, 2005). In parallel, cells were transfected to express wt Arf1 or the DN-Arf1 mutant and infected with FMDV or BEV and processed for confocal microscopy (Fig. S1, available in JGV Online). Infected cells were quantified by labelling for virus. To account for possible DN effects resulting from overexpression of the wt protein, the level of infection for the cells positive for an Arf1 transgene was normalized to the non-expressing cells of the same coverslip. Fig. 4(e) shows that infection by BEV was inhibited by >90 % by DN-Arf1. The wt protein also inhibited BEV infection, although to a lesser extent, possibly due to a dominant-negative effect resulting from overexpression of Arf1. In contrast, wt Arf1 had no effect on FMDV infection, while the DN mutant appeared to enhance infection (Fig. 4e). The observations that DN-Arf1 inhibits BEV but not FMDV infection are consistent with the differential sensitivity of these viruses to BFA. Furthermore, our results suggested that like for BFA treatment, inhibition of Arf1 favours FMDV replication.

### FMDV infection is enhanced by DN-Rab1

Rab1 exists as two isoforms (Rab1a and Rab1b) that are thought to be largely functionally redundant in the early secretory pathway (Tisdale *et al.*, 1992), while Rab1a is also reported to play a role in early-endosome-to-Golgi trafficking (Mukhopadhyay *et al.*, 2011; Sclafani *et al.*, 2010) and autophagy (Winslow *et al.*, 2010). In the early secretory pathway, Rab1 is required for membrane recruitment of GBF1 (Dumaresq-Doiron *et al.*, 2010; Monetta *et al.*, 2007; Nuoffer *et al.*, 1994; Schwartz *et al.*, 2007) and vesicular transport between the ER and Golgi compartments (Allan *et al.*, 2000; Bannykh *et al.*, 2005; Monetta *et al.*, 2007; Pind *et al.*, 1994; Plutner *et al.*, 1990). Consequently, in cells expressing DN mutants of Rab1, COPI assembly is compromised and the Golgi disrupted (Alvarez *et al.*, 2003; Nuoffer *et al.*, 1994; Pind *et al.*, 1994; Plutner *et al.*, 1991; Tisdale *et al.*, 1992). Rab6 functions in multiple Golgi trafficking pathways (Girod *et al.*, 1999; Young *et al.*, 2005) and regulates trafficking within the Golgi cisterna and post-Golgi compartments (Grigoriev *et al.*, 2007). Consequently, in cells expressing DN mutants of Rab6, the Golgi is not disrupted (Martinez *et al.*, 1997; White *et al.*, 1999).

**Fig. 5.**
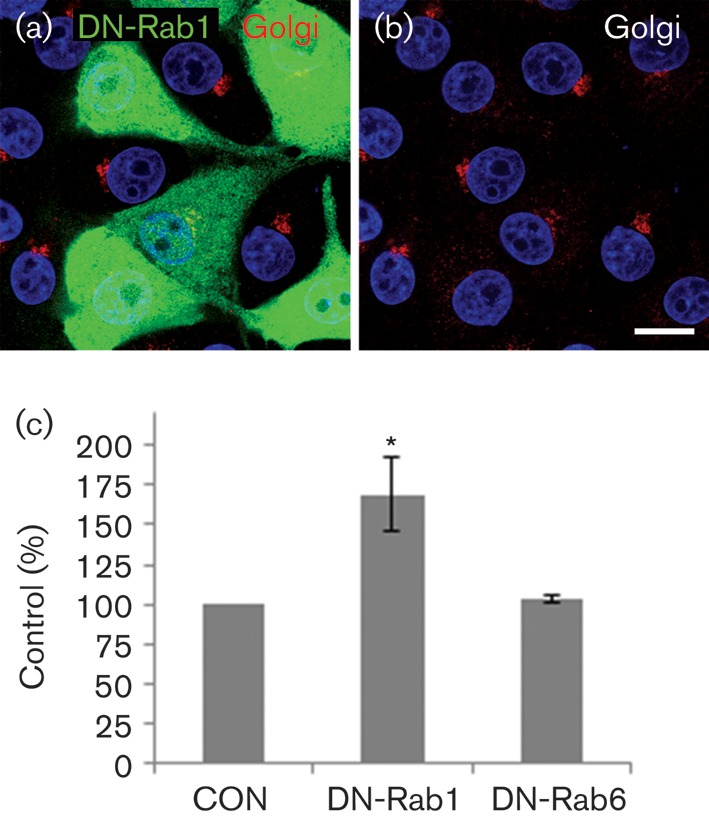
FMDV infection is enhanced by DN-Rab1. IBRS2 cells were transfected with either DN-Rab1a (myc-DN-Rab1a^S25N^) or DN-Rab6 (myc-DN-Rab6^T27N^). (a) Cells transfected with myc-DN-Rab1a^S25N^ labelled for the myc tag (green) and the Golgi (giantin, red). (b) The same cells as in (a) showing Golgi labelling only. The cell nuclei are shown in blue. Bars, 10 μm. (c) Cells transfected with myc-DN-Rab1a^S25N^ or myc-DN-Rab6^T27N^ were infected with FMDV (m.o.i. 0.5) for 3.5 h. The level of infection of cells positive for a DN-Rab transgene was normalized to the non-expressing cells (control, CON) of the same coverslip. The data show the mean±sem for two independent experiments, each carried out with triplicate samples (*n*≥500 cells per coverslip). Student’s *t*-test was used to determine statistical significance (*P<0.05).

Using the same approaches as described above for Arf1, we investigated the effects of expression of myc-tagged DN mutants of Rab1a (myc-DN-Rab1a^S25N^) and Rab6 (myc-DN-Rab6^T27N^) on Golgi integrity and FMDV infection. Consistent with previous reports, DN-Rab1a (Fig. 5a, b) but not DN-Rab6 (data not shown) caused a major disruption to the Golgi. Interestingly, similarly to DN-Arf1, expression of DN-Rab1a enhanced FMDV infection, whereas DN-Rab6 had little or no effect (Figs 5c and S2). Thus, DN mutants of both Arf1 and Rab1a, which are known to inhibit COPI vesicle formation and cause major disruption to the Golgi in IBRS2 cells, appear to enhance FMDV infection. These results suggest that, when membrane flow through the early secretory pathway is arrested before the Golgi, FMDV infection is made more favourable.

### Sar1 is required for FMDV infection

The above results suggest that pre-Golgi membranes of the secretory pathway may be required for FMDV replication as infection is enhanced by reagents (BFA, and DN-Arf1 and Rab1a) that cause Golgi disruption. The first event in the secretory pathway is the generation of COPII vesicles at ERESs. Sar1 is an essential component of COPII and is activated by Sec12, which is insensitive to BFA. Therefore, we investigated the role of Sar1 in FMDV infection using transient transfection of Sar1 mutants and Sar1-targeted siRNA. IBRS2 cells were transfected to express cyan fluorescent protein (CFP)-tagged, wt Sar1a (CFP-wt-Sar1a), DN-Sar1a (CFP-DN-Sar1a^T39N^) or dominant-active (DA) Sar1a (CFP-DA-Sar1a^H79G^). The DN mutant is GDP restricted and blocks the secretory pathway by inhibiting formation of COPII coats and hence COPII-dependent ER export (Barlowe *et al.*, 1994; Kuge *et al.*, 1994). The DA mutant is GTP-bound and stabilized in its active conformation and supports formation of COPII-coated vesicles but arrests further transport by preventing disassembly of the COPII coat (Bielli *et al.*, 2005; Stephens *et al.*, 2000; Ward *et al.*, 2001).

**Fig. 6.**
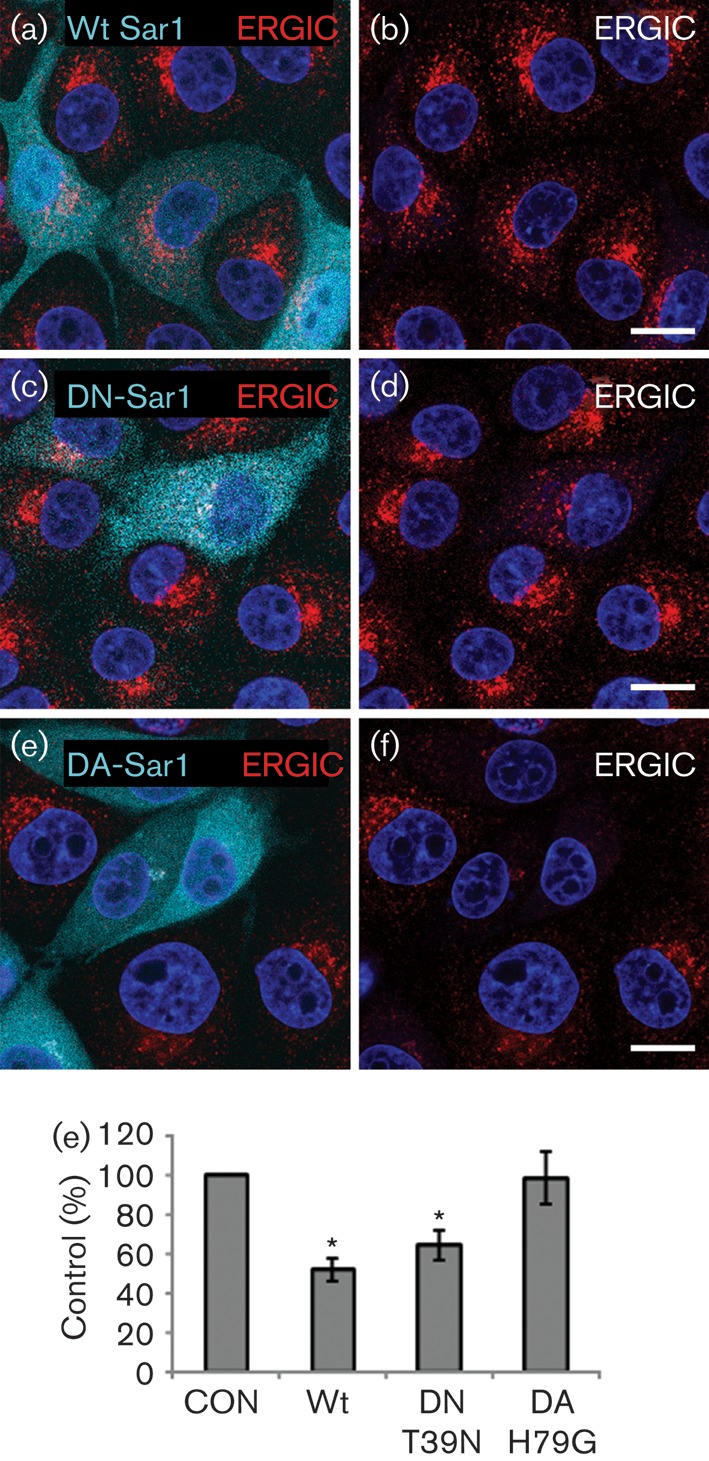
DN-Sar1 but not DA-Sar1 inhibits FMDV infection. IBRS2 cells were transfected with wt Sar1a (CFP-wt-Sar1a), DN-Sar1a (CFP-DN-Sar1a^T39N^) or DA-Sar1a (CFP-DA-Sar1a^H79G^). (a) Cells transfected with wt Sar1a (cyan) labelled for the ERGIC (ERGIC-53, red). (b) The same cells as in (a) showing ERGIC labelling only. (c) Cells transfected with DN-Sar1a (cyan) labelled for the ERGIC (ERGIC-53, red). (d) The same cells as in (c) showing ERGIC labelling only. (e) Cells transfected with DA-Sar1a (cyan) labelled for the ERGIC (ERGIC-53, red). (f) The same cells as in (e) showing only ERGIC labelling. The cell nuclei are shown in blue. Bars, 10 μm. (g) Cells were transfected with wt Sar1a, DN-Sar1a or DA-Sar1a and infected with FMDV (m.o.i. 0.5) for 3.5 h. The level of infection of cells positive for a Sar1 transgene was normalized to the non-expressing cells (control, CON) of the same coverslip. The data show the mean±sem for at least three independent experiments, each carried out with triplicate samples (*n*≥500 cells per coverslip). Student’s *t*-test was used to determine statistical significance (*P<0.05).

First, we examined the effect of DN-Sar1a and DA-Sar1a on the integrity of the ERGIC. Wt Sar1a did not cause notable changes to the ERGIC (Fig. 6a, b), whereas expression of DN-Sar1a caused a partial disruption (Fig. 6c, d). The degree of disruption appeared to vary as, in cells expressing a lower level of DN-Sar1a, the ERGIC remained largely intact. In contrast, expression of DA-Sar1a caused major disruption to the ERGIC (Fig. 6e, f). Parallel transfections were infected with FMDV (Fig. S3) and the effects on infection quantified as described for Arf1. Expression of DN-Sar1a had an inhibitory effect on FMDV infection (Fig. 6g). Wt Sar1a inhibited infection to a similar extent to the DN protein, suggesting that overexpression of wt Sar1a also had a DN effect. In contrast, despite resulting in major disruption to the ERGIC, the DA-Sar1a mutant did not appear to inhibit infection (Fig. 6g). These results suggest that Sar1 is required for FMDV infection and that infection does not require the ERGIC to be intact.

**Fig. 7.**
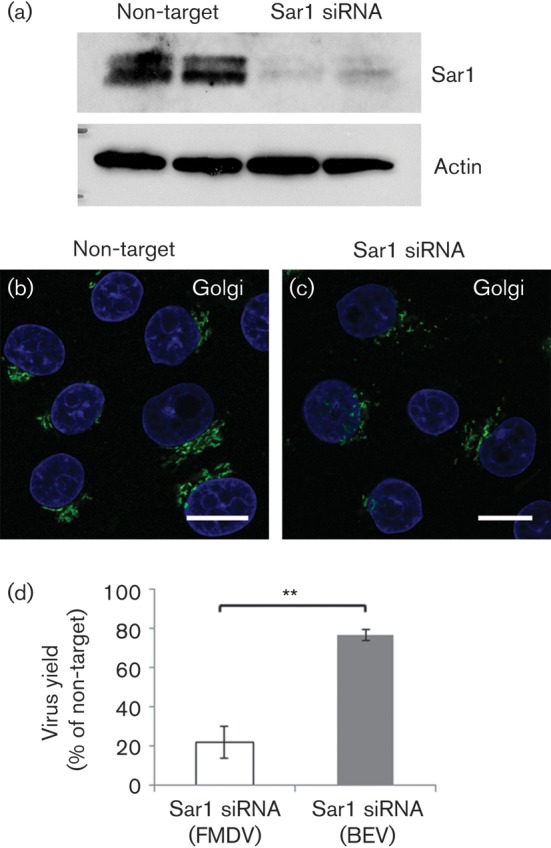
Sar1siRNA inhibits FMDV infection. IBRS2 cells were transfected with non-target siRNA or Sar1-targeted siRNA. (a) Western blot for Sar1 and actin (loading control). In the Sar1 blot, the upper band is Sar1b and the lower band Sar1a. Shown are the representative results for duplicate transfections. (b) Cells transfected with non-target siRNA labelled for the Golgi (GM130, green). (c) Cells transfected with Sar1 siRNA and similarly labelled for the Golgi. Cell nuclei are shown in blue. Bars, 10 μm. (d) IBRS2 cells were transfected with non-target or Sar1-targeted siRNA and infected with either FMDV or BEV (m.o.i. 1.0). Shown is the virus yield at 4 h p.i. for the cells transfected with Sar1 siRNA as a percentage of virus yields for the cells transfected with the non-target siRNA. Open bars show data for FMDV; grey bars show data for BEV. The data show the mean±sem for three independent experiments, each carried out with triplicate samples. Student’s *t*-test was used to determine statistical significance (**P<0.01).

To confirm the role of Sar1 in FMDV infection, cells were depleted of Sar1 using siRNA. Sar1 is expressed as two isoforms, Sar1a and Sar1b. IBRS2 cells were transfected for 48 h with siRNA to Sar1a and Sar1b and the knockdown confirmed by Western blotting. Fig. 7(a) shows that Sar1-targeted siRNA caused a large reduction in Sar1 protein. Analysis of the Golgi in siRNA transfected-cells showed that the control non-targeted siRNA had no effect on the Golgi (Fig. 7b), while the Golgi was disrupted in cells transfected with Sar1 siRNA (Fig. 7c). The Golgi was disrupted in ~75 % of the cells, which is consistent with the transfection efficiency as determined using siGLO reagents (data not shown).

Cells transfected with the non-target or Sar1 siRNAs were also infected with FMDV or BEV. At 4 h post-infection (p.i.), the supernatants were collected and the amount of infectious virus present determined by plaque assay. The virus yield for cells transfected with the Sar1 specific siRNA was reduced by ~80 % for FMDV and by ~20 % for BEV when compared with cells transfected with the control, non-targeted siRNA (Fig. 7d). The cells from these experiments were fixed and processed for confocal microscopy to quantify the number of infected cells (Fig. S4). The level of infection was similar (~50 % infection; *n*≥300) for cells transfected with non-targeted control siRNA or Sar1-specific siRNA for both FMDV and BEV (data not shown), indicating that Sar1 knockdown had not affected cell entry. These results showed that Sar1 is required for the post-entry phase of FMDV replication.

### FMDV infection leads to dispersal and reduction of Sec31 labelling

**Fig. 8.**
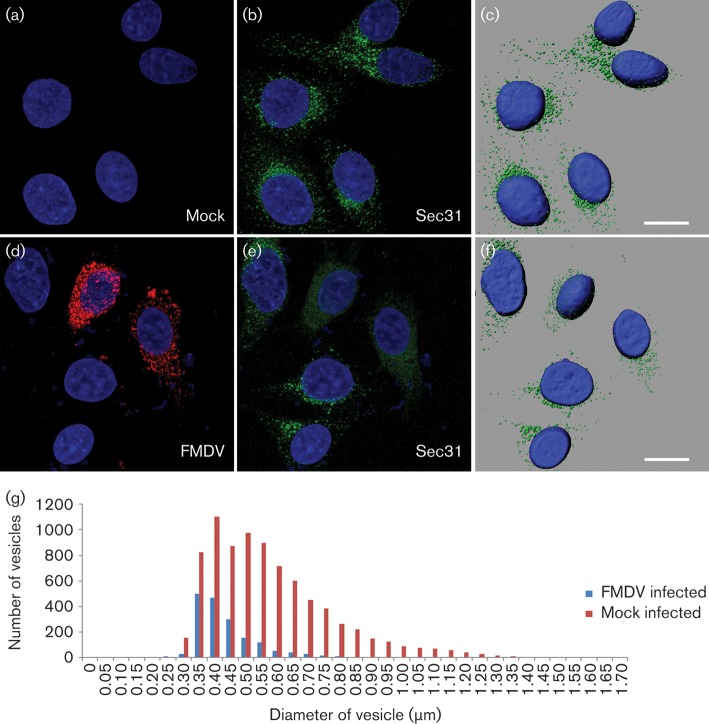
FMDV infection leads to dispersal and reduction of Sec31 labelling. HeLa cells were mock infected or infected with FMDV O1BFS/1860 (m.o.i 0.5) for 3 h. (a, b) Cell nuclei (a) and Sec31 labelling (green) (b) in mock-infected cells. (c) A rendered image of the cells shown in (b). (d) FMDV (labelled for 3A; red) and (e) Sec31 labelling (green) in infected cells. (f) A rendered image of the cells shown in (e). The cell nuclei are shown in blue. Bars, 10 μm. The IMARIS spot function was used to identify Sec31-positive punctae in 35 mock-infected and 35 infected cells. (g) Sec31 punctae size plotted against frequency.

Enteroviruses subvert Arf1-dependent COPI vesicle formation for replication but exclude COPI coat components from the replication complex (Hsu *et al.*, 2010). Therefore we determined the location of the outer COPII coat protein, Sec31, in infected cells. We were unable to obtain labelling for Sec31 in IBRS-2 cells due to poor cross-reactivity of the antibody. Therefore, we used HeLa cells and FMDV O1BFS/1860, which infects cells using heparan sulphate receptors (Jackson *et al.*, 1996). Infection of HeLa cells by FMDV O1BFS/1860 was productive but showed a delayed cytopathic effect (at ~6–8 h p.i.) compared with IBRS-2 cells (data not shown). The effect of FMDV on Sec31 was examined by confocal microscopy at hourly intervals and infection was indicated by the presence of labelling for the FMDV 3A protein. The 3A protein was not detectable at 1 h p.i. while at 2 h p.i. a small number of cells labelled weakly for 3A (data not shown). At 3 h p.i., over 50 % of the cells contained high levels of FMDV 3A. Prior to 3 h p.i., there was no discernible effect on Sec31 labelling when compared with mock-infected cells (data not shown). However, at 3 h p.i., most of the infected cells showed an apparent decrease in the number and size of Sec31-positive punctae (Fig. 8). At this time point, the ER remained intact (data not shown). The IMARIS spot function (see Methods) was used to quantify Sec31-positive punctae in 35 mock-infected and 35 infected cells. Fig. 8(g) shows punctae size plotted against frequency and showed that infected cells had a twofold reduction in number of Sec31 punctae and a greater proportion were smaller. Infection of cells by FMDV results in the rapid inhibition of host-cell protein synthesis (Belsham *et al.*, 2000). To examine whether shutoff of protein synthesis leads to disruption of Sec31 labelling, HeLa cells were incubated with cycloheximide (which blocks protein synthesis) (Armer *et al.*, 2008) and the cells examined at 3 h p.i. The levels and location of Sec31 in cycloheximide-treated cells were similar to those of mock-treated cells, indicating that the effects triggered by shutoff of protein synthesis did not lead to disruption of Sec31 (Fig. S5).

Here, we have provided evidence that membranes for FMDV replication derive from pre-Golgi compartments of the early secretory pathway. This conclusion is supported by three key observations. Firstly, inhibition of Sar1 by expression of a DN-Sar1a mutant or by Sar1 depletion appeared to inhibit membrane flow from the ER to the ERGIC (Fig. 6) and Golgi (Fig. 7) and reduced FMDV infection, suggesting that FMDV replication occurs by a Sar1-dependent process. Secondly, when the ERGIC was disrupted by DA-Sar1a, FMDV infection was not inhibited. The DA mutant supports formation of COPII-coated vesicles but arrests further transport by preventing disassembly of the COPII coat. This suggests that FMDV is either exploiting COPII directly or using membrane deformation generated by COPII assembly to promote infection. Finally, BFA and DN mutants of Arf1 and Rab1a, which are known to block the early secretory pathway at the pre-Golgi stage (Allan *et al.*, 2000; Dascher & Balch, 1994; Lippincott-Schwartz *et al.*, 1989), were shown to disrupt the Golgi in IBRS-2 cells (Figs 2, 4 and 5) and enhance FMDV infection. Presently, it is not clear how these reagents make FMDV infection more likely; however, they are known to inhibit the early secretory pathway at effectively the same step (i.e. immediately after COPII vesicle budding) and could benefit FMDV replication by restricting membrane flow through the ERGIC and Golgi, thereby providing more membranes for viral replication complex formation. Similar observations have been made by Coyne *et al.* (2011) who observed that a greater proportion of cells were infected by CVB and PV when the functions of specific cellular proteins had been compromised by siRNA depletion.

Recently, PV has been reported to transiently stimulate the production of COPII vesicles during the early phase of infection, which is followed by a subsequent inhibition (Trahey *et al.*, 2012). Although we did not observe differences in labelling for Sec31 at earlier time points (i.e. 1 and 2 h p.i.), we did see a reduction in Sec31 labelling at 3 h p.i. (Fig. 8). This was coincident with the detection of the viral 3A protein, which probably indicates that Sec31 labelling is reduced at a time when replication complexes are being formed. The reduction in Sec31 labelling suggests that ERES may be compromised; however, this may not necessarily be the case, as the production of membrane-bound vesicles from the ER may continue in FMDV-infected cells with the possibility that the outer COPII coat components (e.g. Sec31) are excluded from the replication complex. This would be consistent with enteroviruses, which subvert COPI vesicle production for replication but exclude COPI components from the replication complex (Hsu *et al.*, 2010). Aichi virus (genus *Kobuvirus*, family *Picornaviridae*) has been shown to recruit PI4K to replication membranes using a different strategy to that employed by PV (see Introduction). For Aichi virus, recruitment of PI4K is dependent on ACBD3 (acyl-coenzyme A-binding domain containing 3) and not GBF1/Arf1 which could explain the BFA insensitivity of this virus. Further studies will be required to determine if PI4K and ACBD3 are required for FMDV infection and to define more precisely the cellular origin of FMDV replication membranes.

## Methods

### 

#### Cells and viruses.

IBRS-2 cells were cultivated in Glasgow’s modified Eagle’s medium with 10 % adult bovine serum, BHK cells in Dulbecco’s modified Eagle’s medium with 5 % FCS, and HeLa cells in HEPES-buffered Dulbecco’s modified Eagle’s medium with 10 % FCS. All media were supplemented with 20 mM l-glutamine, 100 SI units penicillin ml^−1^, and 100 µg streptomycin ml^−1^. Working stocks of FMDV O1Kcad2 and O1BFS/1860 were prepared as described previously (Berryman *et al.*, 2005; Jackson *et al.*, 1996). Working stocks of BEV-1 were prepared using IBRS2 cells. The m.o.i. was based on the virus titre on IBRS2 cells, as described previously (Jackson *et al.*, 2000).

#### Antibodies and reagents.

The anti-c-myc antibody (9E10) was from the Developmental Studies Hybridoma Bank, (University of Iowa, IA, USA) and the anti-haemagglutinin (anti-HA) from Roche. Mouse anti-Sec31A was from Transduction Laboratories. Antibodies to the Golgi markers were to GM130 (BD Transduction Laboratories) and Giantin (Millipore). ERGIC labelling used anti-ERGIC-53 (Sigma). ER labelling used anti-ERp57 (Rouiller *et al.*, 1998). Anti-Sar1 and anti-actin were from Millipore and Sigma, respectively. Species-specific, Alexa Fluor (488, 568 and 633) conjugated secondary antibodies were from Life Technologies and anti-mouse/rabbit HRP secondary antibodies were from Promega. FMDV-infected cells were detected using a rabbit polyclonal serum generated using whole virus as the immunogen or the mAb 2C2, which recognizes the viral 3A protein (De Diego *et al.*, 1997; Reid *et al.*, 2009). BEV-infected cells were detected using a guinea pig polyclonal serum generated using whole virus as the immunogen. BFA (10 mg ml^−1^; ready-made solution) was from Sigma. Cycloheximide was from Oxoid. For experiments using BFA, DMSO (the solvent) was added to mock-treated controls. Expression plasmids were gifts from A. Townley (University of Bristol, UK) – CFP-wt-Sar1a, CFP-DN-Sar1a^T39N^ and CFP-DA-Sa1a^H79G^; E. Ehrenfeld (NIAID, NIH, Bethesda, MD, USA) – GFP-wt-Arf1; J. Lippincott-Schwartz (National Institutes for Health, MD, USA) – HA-DN-Arf1^T31N^; and T. Herbert (McGill University, Montreal, Canada) – Myc-DN-Rab1a^S25N^ and Myc-DN-Rab6^T27N^.

#### Infection of transfected cells.

Cells on glass coverslips were transfected using 1 μg plasmid DNA and Lipofectamine 2000 (Invitrogen) as described previously (Gold *et al.*, 2010) and used for all experiments at 14 h post-transfection. Transfection efficiencies ranged from 20 to 45 %. Transfected cells were infected with FMDV or BEV at the indicated m.o.i. Infection was stopped and the cells fixed using 4 % paraformaldehyde for 40 min. The cells were processed for immunofluorescence confocal microscopy. Cells expressing a transgene were identified by either the fluorescence of CFP or GFP, or by detection of c-myc or HA epitope tags using the appropriate primary and secondary antibodies. For each experiment using Arf1, Sar1 or Rab protein, cells on triplicate coverslips were transfected with each construct and between 500 and 1000 cells scored for infection for both the transfected and non-transfected cell populations using randomly selected fields of view.

#### Immunofluorescence confocal microscopy.

Cells were fixed with paraformaldehyde and processed for confocal microscopy as described previously (Gold *et al.*, 2010). Briefly, cells were permeablized with 0.1 % Triton X-100 and non-specific binding sites blocked. The cells were incubated sequentially for 1 h each with the appropriate primary antibody followed by the appropriate species-specific, Alexa Fluor-conjugated secondary antibody. The cell nuclei were labelled with DAPI. Coverslips were mounted in Vectashield mounting medium for fluorescence. Cells were viewed using a Leica SP2 laser-scanning confocal microscope and optical sections recorded using either the ×63 or ×40 oil-immersion objective with a numerical aperture of 1.4 and 1.25, respectively. The data are shown as single optical sections through the middle of the cell with the exception of Figs 1 and 8, which show maximum projections of z-stacks (14 sections; spacing 0.5 μm). All data were collected sequentially to minimize cross-talk between fluorescent signals. Images were processed using Adobe Photoshop software.

For imaging processing using IMARIS (Bitplane Scientific Software), images were recorded in sequential scanning mode. Three-dimensional datasets of cells labelled for Sec31 and FMDV 3A, and DAPI treated were acquired using Leica SP2 stack function (14 sections; spacing 0.5 μm). Thirty-five infected cells and 35 mock-infected cells were analysed and their Sec31-labelled vesicles were detected with the spot function of IMARIS.

#### siRNA reagents and infection of transfected cells.

siRNA duplexes were from Dharmacon: two target Sar1a (sense strands 5′-CUACAAGAAAUCCGGAAAAUU-3′ and 5′-AGUCGAGCUUAAUGCUUUAUU-3′) and two target Sar1b (sense strands 5′-CAUGAAAGGCUGUUAGAAUUU-3′ and 5′-GCUCGGAGAGUGUGGAAAAUU-3′). Control, non-target AllStars siRNA was from Qiagen. Cells were transfected with control, non-target siRNA (40 pmol) or all four Sar1 siRNAs using 10 pmol each duplex. Cells on glass coverslips were transfected using Lipofectamine 2000 and used at 48 h post-transfection. Transfection efficiencies were determined using siGLO reagent (Thermoscientific) and were consistently greater than 75 %. Transfected cells were infected with FMDV or BEV for 1 h (m.o.i. 0.5). For FMDV, the cells were washed and incubated with low-pH buffer (Berryman *et al.*, 2005) for 3 min to inactivate extracellular virus, followed by washing with cell culture medium. For BEV, the cells were washed extensively (at neutral pH) to remove excess virus. Samples of cell supernatants were collected immediately after washing or after a further 3 h at 37 °C. Virus yields were determined by titration on BHK cells by standard plaque assay (Jackson *et al.*, 2000). At the end of the infection period, the cell monolayers were fixed and processed for confocal microscopy labelling for FMDV or BEV and the percentage of infected cells was determined.

#### Western blotting.

The efficiency of Sar1 knockdown was evaluated by Western blotting. The amount of protein in each sample was determined by BCA assay (Thermo Scientific Pierce). Cell lysates were separated by SDS-PAGE (10 % acrylamide) and transferred to Hybond-C Extra membrane (Amersham), blocked and probed with primary antibodies to Sar1 or actin, followed by the appropriate HRP-conjugated secondary antibody. Signals were developed using enhanced chemiluminescence reagents (Pierce).
